# Genetic drift acts strongly on influenza virus populations within acute human infections but is obscured by other factors within acutely infected swine

**DOI:** 10.1093/ve/veag021

**Published:** 2026-04-01

**Authors:** Yike T Shi, Michael A Martin, Daniel B Weissman, Katia Koelle

**Affiliations:** Department of Biology, Emory University, 1510 Clifton Rd NE #2006, Atlanta, GA 30322, United States; Department of Ecology and Evolutionary Biology, 106A Guyot Ln, Princeton, NJ 08544, United States; Department of Pathology, Johns Hopkins School of Medicine, 600 N. Wolfe Street, Baltimore, MD 21287, United States; Department of Epidemiology, Johns Hopkins Bloomberg School of Public Health, 615 N. Wolfe Street, Baltimore, MD 21205, United States; Department of Biology, Emory University, 1510 Clifton Rd NE #2006, Atlanta, GA 30322, United States; Department of Physics, Emory University, 400 Dowman Dr, Atlanta, GA 30322, United States; Department of Biology, Emory University, 1510 Clifton Rd NE #2006, Atlanta, GA 30322, United States; Emory Center of Excellence for Influenza Research and Response (CEIRR), 1510 Clifton Rd NE, Atlanta, GA 30322, United States

**Keywords:** intrahost viral evolution, influenza A virus evolution, genetic drift, sampling noise, stochastic processes

## Abstract

The evolutionary dynamics of seasonal influenza A viruses (IAVs) have been well characterized at the population level, with antigenic drift known to be a major force in driving strain turnover. The evolution of IAV populations at the within-host level, however, is still less well characterized. Improving our understanding of within-host IAV evolution has the potential to shed light on the sources of new strains, including new antigenic variants, at the population level. Existing studies have pointed towards the role that stochastic processes play in shaping within-host viral evolution in acute infections of both humans and pigs. Here, we first apply a population genetic model called the “Beta-with-Spikes” approximation to longitudinal intrahost single-nucleotide variant (iSNV) frequency data to quantify the extent of genetic drift acting on IAV populations at the within-host scale. We estimate a small effective population size for human IAV infections ($N_{E} = 49$, 95%CI: [28, 84]) and show that the observed iSNV dynamics are consistent with a Wright–Fisher model using various summary statistics. Using a diffusion approximation approach, we then further show that sampling noise is small relative to the magnitude of genetic drift in this dataset. We then apply similar analyses to the swine IAV dataset, arriving again at a very small effective viral population size estimate. However, we find that features of the swine IAV data cannot be consistently accounted for with a basic Wright–Fisher model of evolution and that sampling noise (in the broadest sense) can better account for the iSNV frequency changes observed in the swine IAV data. Our findings on IAV evolution within acutely infected humans contribute to a growing number of studies that point towards the important role of genetic drift in shaping patterns of genetic diversity in this host. Our findings also raise questions about what processes (e.g. spatial within-host compartmentalization, ecological superinfection) may impede our ability to quantify the strength of genetic drift acting on IAV populations in acutely infected swine.

## Introduction

Viral adaptation at the population level ultimately depends on genetic variation, i.e. generated during viral replication at the within-host scale. Several different mechanisms, however, can underlie the path from mutation generation to spread at the population level. One possibility is that advantageous mutations that are generated during replication (e.g. those altering the antigenicity of a virus) are efficiently selected at the within-host scale, and then subsequently spread at the level of the population. Alternatively, selection may be inefficient at the within-host scale, with population-level spread of advantageous mutations occurring largely due to selection at this higher organizational scale. A previous analysis of synonymous and nonsynonymous viral genetic variation within hosts and at the population level found strong support for the latter mechanism in human influenza viruses, with both purifying selection and positive selection (at antigenic sites) acting more strongly at the population level than at the within-host level (Xue and Bloom 2020). Consistent with this finding, earlier within-host studies indicated that influenza A virus (IAV) diversity was limited in acutely infected individuals and that the diversity that was observed was largely shaped by genetic drift and purifying selection (Dinis et al. 2016; Debbink et al. 2017; McCrone et al. 2018). Positive selection on within-host IAV populations was not detected in any of these studies. Similar results to these were found for within-host IAV populations in swine hosts experiencing acute infection (VanInsberghe et al. 2024).

Using serial samples from 43 acutely infected individuals from the McCrone et al. (2018) study, McCrone et al. (2020) applied a population genetic model to human influenza A intrahost single nucleotide variant (iSNV) data to quantify the strength of genetic drift in these within-host viral populations. Relying on a diffusion approximation, this analysis estimated a very small effective population size ($N_{E}$), on the order of 32–72 virions. A more recent analysis of IAV evolution using a different dataset with serial samples from 143 acutely infected individuals estimated within-host $N_{E}$ values of 176–284 using approximate Bayesian computation (ABC) (Bendall et al. 2024). This study also found evidence for positive selection occurring at 9%–11% of the variable sites. As such, findings from this recent analysis differ to some extent from the previous ones, finding lower levels of genetic drift (higher $N_{E}$) and some evidence of positive selection. Finally, a study of within-host IAV evolution in young children found evidence for low viral diversity and purifying selection in seasonal H3N2 infections early on in the course of infection and accumulation of nonsynonymous variants starting around 3–4 days post-symptom onset (Han et al. 2021).

Here, similarly to some of the above studies, we aim to quantify the effective population size $N_{E}$ of within-host IAV populations. Moreover, we ask whether the evolutionary dynamics observed in within-host IAV populations are consistent with the classic Wright–Fisher model of evolution. We first quantify $N_{E}$ using McCrone et al. (2018) previously published within-host IAV data from humans. To quantify $N_{E}$, however, we use a different population genetic model, namely the “Beta-with-Spikes” model (Tataru et al. 2015). We use this model due to its demonstrated ability to capture the distribution of allele frequencies (DAFs) over time under a Wright–Fisher model with both large and small population sizes. In contrast, the diffusion approximation is only considered a good approximation when effective population sizes are large. Our analyses support very small within-host viral effective population sizes in acutely infected humans. Furthermore, we find that within-host IAV evolutionary dynamics observed in humans are consistent with dynamics arising from the classic Wright–Fisher model and that sampling noise is low in this dataset. We then apply analogous analyses to a previously published within-host IAV data from swine (VanInsberghe et al. 2024). For this dataset, we instead find that the classic Wright–Fisher model cannot reproduce key features of the observed iSNV frequency changes. Instead, we find that sampling noise (in the broadest sense) dominates observed patterns in iSNV frequency changes in this dataset. We discuss potential biological factors at play in contributing to this noise and argue that these factors impede our ability to quantify the extent of genetic drift acting on swine IAV populations in this dataset.

## Methods

### Overview of the Beta-with-Spikes model

We estimate the effective population size $N_{E}$ of within-host IAV populations using a population genetic model called the Beta-with-Spikes model (Tataru et al. 2015). This model approximates the DAFs that would result from a Wright–Fisher model over discrete generations. As its name implies, the model uses an adjusted form of the beta distribution. The adjusted form of this distribution includes two spikes at frequencies of 0.0 and 1.0 that account for the probabilities of loss and fixation of alleles, respectively. The DAFs under the Beta-with-Spikes model in generation $t$ is given by equation (8) in Tataru et al. (2015), reproduced here: 


(1) \begin{align*}& \begin{split} f^\star_{B}(x;t) =\ & \mathbb{P}(X_{t} = 0) \cdot \delta(x) + \mathbb{P}(X_{t} = 1) \cdot \delta(1-x) \\ & + \mathbb{P}(X_{t} \notin \{0,1\}) \cdot \frac{x^{\alpha^\star_{t} - 1} (1-x)^{\beta^\star_{t} - 1}}{B(\alpha^\star_{t}, \beta^\star_{t})} \end{split}\end{align*}

where $\delta (x)$ is the Dirac delta function. The three terms on the right-hand side of this equation correspond to the probability mass of allele loss, the probability mass of allele fixation, and the probability densities of allele frequencies between (but excluding) 0 and 1, respectively. The calculation for the beta distribution’s shape parameters $\alpha ^\star _{t}$ and $\beta ^\star _{t}$ as well as the probabilities for loss and fixation for each generation $t$ can be found in Tataru et al. (2015).

**Figure 1:**
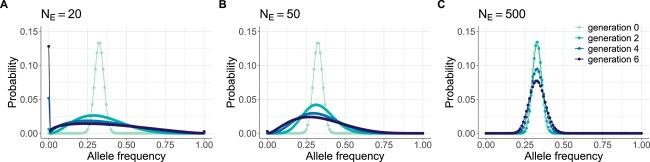
Simulated DAFs under the Beta-with-Spikes approximation for the Wright–Fisher model. Panels (A)–(C) provide simulations under different effective population sizes: (A) $N_{E} = 20$, (B) $N_{E} = 50$, (C) $N_{E} = 500$. In all panels, the initial iSNV frequency distribution in generation 0 was parameterized with a mean value of $p_{0} = 0.325$ and variance given by $v_{0} = m p_{0} (1-p_{0})$, with $m=0.004$. DAFs from generations 2, 4, and 6 are plotted in each panel to show changes in DAFs across generations. The DAFs in panels (A)–(C) are shown by plotting probability masses in frequency bins having widths of 0.01. The values used for $p_{0}$ and $m$ for the DAF simulations shown in this figure were chosen for purely illustrative reasons. A mean initial frequency closer to 0.5 yields more distinct DAFs between different $N_{E}$ values and across generations. However, using a mean initial frequency further away from 0.5 results in patterns of loss and fixation that are more asymmetric. We therefore used an initial frequency of $p_{0} = 0.325$. The value of $m$ used corresponds to the value of $m$ used in our applications to the human and swine IAV datasets. The binomial form for the variance has the desirable property that the variance is 0 at the boundary frequencies of $p_{0} = 0$ and $p_{0} = 1$ and highest at a frequency of $p_{0} = 0.5$. Furthermore, this binomial form is consistent with a sampling process, i.e. noisy, with $m$ quantifying the extent of sampling noise.

For the reader to develop familiarity with evolutionary dynamics under the Beta-with-Spikes model, we show in Fig. 1 simulated DAFs under this model for three different effective population sizes, each starting with the same DAF in generation 0. Figure 1A shows how the DAF changes when the effective population size $N_{E}$ is very small ($N_{E} = 20$): the distribution rapidly spreads out from its initial distribution within a small number of generations. By generation 6, allele loss ($X_{t} = 0$) already accounts for upwards of 12% of the probability mass. At larger values of $N_{E}$ (Fig. 1B and C), the DAF spreads out from its initial distribution more slowly, as anticipated. For example, when $N_{E} = 500$, allele loss and fixation accounts for <1% of the probability mass by generation 6 (Fig. 1C).

### Within-host human influenza A virus data

We first analyzed a previously published human IAV dataset (McCrone et al. 2018), sourced from a community-based cohort study. This deep-sequencing dataset contains samples from 49 individuals. Forty three of these 49 individuals were longitudinally sampled, with two samples that were collected between −2 and 6 days post symptom onset. A subset of the samples from the 49 individuals had technical sequencing replicates available. For those samples with technical replicates, we used the replicate that had higher sequencing depth. Variants were called at a minor allele frequency threshold of 2% from sequencing reads accessed through the NCBI Sequencing Read Archive (NCBI SRA BioProject PRJNA412631). For each of the 43 longitudinally sampled individuals, Supplemental Table S1 lists the two sample collection dates (relative to symptom onset day) and the identities and frequencies of all of the identified iSNVs. Only 37 of these 43 individuals had iSNVs that exceeded frequencies of 2%.

**Figure 2:**
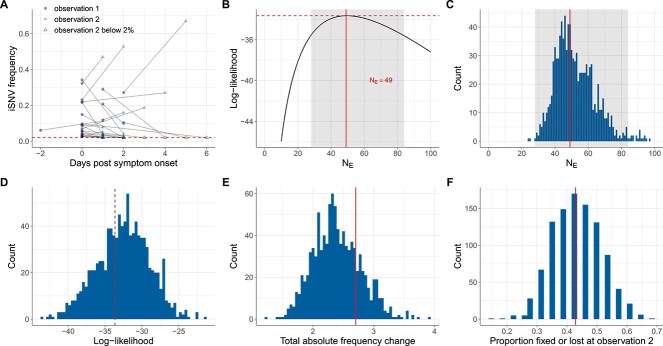
Estimates of within-host IAV effective population size from iSNVs present in the first sampled timepoint (human data subset 1) and evaluation of the consistency of these data with the Wright–Fisher model. (A) Allele frequency changes between the first observation time point and the second observation time point. Allele frequencies are plotted by day of symptom onset of the infected individual. The dashed line shows the variant-calling threshold of 2%. Allele frequencies that fall under this threshold are shown at the threshold. (B) Calculated log-likelihood values across a range of effective population sizes. Solid line shows the MLE of $N_{E}$. Dashed line shows the log-likelihood value for the MLE of $N_{E}$. The shaded region shows the 95% CI around the MLE of $N_{E}$. (C) Maximum-likelihood estimates of $N_{E}$ from the 1000 simulated datasets, obtained using the Beta-with-Spikes model (histogram). The MLE of $N_{E} = 49$ from human data subset 1 is shown with a solid line. The shaded region shows the 95% CI shown in panel B. (D) Calculated log-likelihood values for 1000 mock iSNV datasets that were generated by forward simulation of the Beta-with-Spikes model with $N_{E} = 49$ (histogram). The log-likelihood value at $N_{E} = 49$ calculated from human data subset 1 is shown with a dashed line. (E) Distribution of total absolute frequency changes ($\sum |\Delta f|$) for the 1000 mock iSNV datasets (histogram). Solid line shows the total absolute frequency change calculated from human data subset 1. (F) Distribution of the proportion of iSNVs either lost or fixed at the second time point for the 1000 mock iSNV datasets (histogram). Solid line shows the proportion of iSNVs lost or fixed at the second time point calculated from human data subset 1.

We perform two separate analyses, on different subsets of these data. We first estimate $N_{E}$ using the subset of iSNVs that were detected above our variant-calling threshold of 2% at the first of the two sample collection time points. This includes iSNVs that were still detected above 2% at the second time point as well as iSNVs that were no longer detected or fell below 2% at the second time point. To avoid bias that could result from genetic linkage, we downsample this set of iSNVs to one iSNV per individual. We do this by selecting the iSNV that has a frequency closest to 50% at the first time point and should therefore be most informative of $N_{E}$. The downsampled subset of iSNVs comprises 28 paired-time observations. We refer to this dataset as human IAV data subset 1. Supplemental Table S1 indicates the iSNVs that are included in data subset 1 and Fig. 2A plots the frequency dynamics of these data subset 1 iSNVs. As a sensitivity analysis, we also consider random downsampling of iSNVs.

**Figure 4:**
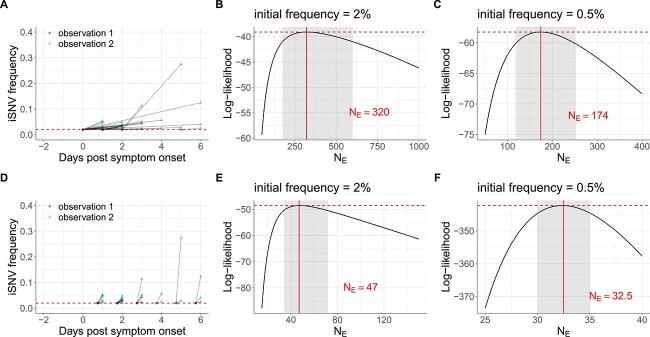
Estimates of within-host IAV effective population sizes from iSNVs present only in the second sampled timepoint (human data subset 2). (A) Allele frequencies at the first and second observation time points in human data subset 2. As in Fig. 2A, iSNV frequencies are plotted according to the day of symptom onset and the dashed red line shows the variant-calling threshold of 2%. Below-the-variant-calling-threshold iSNV frequencies at the first time point are shown at 2%. (B) Calculated log-likelihood values across a range of effective population sizes when iSNVs at the first observation time point are assumed to be present at a frequency of 2%. (C) Calculated log-likelihood values across a range of effective population sizes when iSNVs at the first observation time point are assumed to be present at a frequency of 0.5%. (D) Allele frequencies from the paired-time observations in human data subset 2, assuming that the iSNV frequencies were present at the variant-calling threshold a single viral generation prior to the second observation time point. As in Fig. 4A, iSNV frequencies are plotted according to the day of symptom onset and the dashed red line shows the variant-calling threshold of 2%. (E) Calculated log-likelihood values across a range of effective population sizes for the modified human data subset 2 shown in panel (D). (F) As in panel (E), calculated log-likelihood values across a range of effective population sizes when iSNVs at the first time point are instead assumed to be present at a frequency of 0.5%. In panels (B), (C), (E), and (F), the solid red lines show the MLE of $N_{E}$ and dashed red lines show the log-likelihood value for the MLE of $N_{E}$. The shaded regions show the 95% CI around the MLE of $N_{E}$.

Our second analysis estimates $N_{E}$ using the subset of iSNVs that were detected above our variant-calling threshold of 2% at the second time point but were either undetected or fell below 2% at the first of the two time points. To again avoid bias that could result from genetic linkage, we downsample this set of iSNVs to one per individual, this time selecting the iSNV we keep in our dataset at random. The downsampled subset of iSNVs comprises 30 paired-time observations. We refer to this dataset as human IAV data subset 2. Supplemental Table S1 indicates the iSNVs that are included in data subset 2 and Fig. 4A plots the frequency dynamics of these data subset 2 iSNVs. Our partitioning of the human IAV data into these two subsets allows us to avoid estimation of the mutation rate. Estimation of the mutation rate is difficult because mutation rates affect not only the frequencies of detected iSNVs but also how often one might expect polymorphic sites to arise, which would require integration of data from all the other nucleotide sites.

Finally, for some of our analyses below, we make use of samples taken from the same individual on the same day. These samples are biological replicates, where the first sample was self-taken by the study participant and the second sample was taken in a follow-up clinic visit on the same day. Supplemental Table S2 lists the iSNVs in this dataset.

### Within-host swine influenza A virus data

We further analyzed a previously published swine IAV dataset (VanInsberghe et al. 2024), sourced from a week-long county fair. This dataset includes deep-sequencing data from 76 longitudinally sampled pigs with single-subtype infections. Approximately 6% of the sampled pigs had >2 samples taken (range: 3–5 samples). Variants were called from sequencing reads accessed through NCBI SRA (BioProject PRJNA1051292) with a variant calling threshold of 2%, as for the human IAV infections.

**Figure 5:**
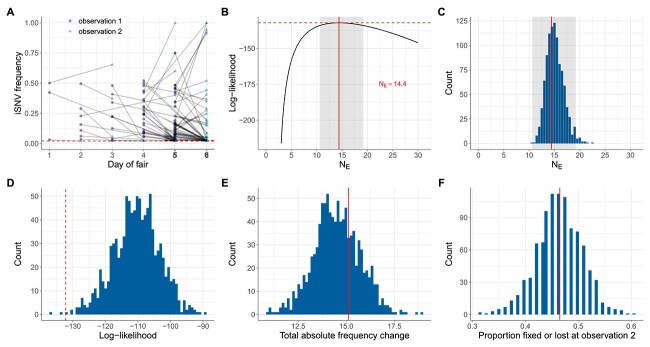
Estimates of within-host IAV effective population size from iSNVs present in swine data subset 1 and evaluation of the consistency of these data with the Wright–Fisher model. (A) Allele frequency changes between the first observation time point and the second observation time point. Allele frequencies are plotted by day of county fair. The dashed line shows the variant-calling threshold of 2%. Allele frequencies that fall under this threshold are shown at the threshold. (B) Calculated log-likelihood values across a range of effective population sizes. Solid line shows the MLE of $N_{E}$. Dashed line shows the log-likelihood value for the MLE of $N_{E}$. The shaded region shows the 95% CI around the MLE of $N_{E}$. (C) MLEs of $N_{E}$ from 1000 simulated datasets, obtained using the Beta-with-Spikes model (histogram). The MLE of $N_{E} = 14.4$ from swine data subset 1 is shown with a solid line. The shaded region shows the 95% CI shown in panel B. (D) Calculated log-likelihood values for 1000 mock iSNV datasets that were generated by forward simulation of the Beta-with-Spikes model with $N_{E} = 14.4$ (histogram). The log-likelihood value at $N_{E} = 14.4$ calculated from swine data subset 1 is shown with a dashed line. (E) Distribution of total absolute frequency changes ($\sum |\Delta f|$) for the 1000 mock iSNV datasets (histogram). Solid line shows the total absolute frequency change calculated from swine data subset 1. (F) Distribution of the proportion of iSNVs either lost or fixed at the second time point for the 1000 mock iSNV datasets (histogram). Solid line shows the proportion of iSNVs lost or fixed at the second time point calculated from swine data subset 1.

We generated analogous datasets to human data subset 1 and human data subset 2 for these swine IAV data, using similar approaches as those described above. We refer to these swine datasets as swine data subset 1 and swine data subset 2, respectively. For swine data subset 1, we downsampled the eligible iSNVs to one iSNV per pair of adjacent time points by selecting the iSNV with frequency closest to 50% at the first time point of the pair. For swine data subset 2, we downsampled the set of iSNVs analyzed to one iSNV per pair of adjacent time points by selecting an iSNV from the eligible set of iSNVs at random. In all, swine data subsets 1 and 2 each include 86 paired-time observations. Supplemental Table S3 lists the identified iSNVs in the swine data and which iSNVs are included in each of the two swine data subsets. Figure 5A plots the frequency dynamics of the iSNVs in swine data subset 1.

### Bioinformatic processing

We reanalyzed the sequence data that were made publicly available in McCrone et al. (2018) and VanInsberghe et al. (2024) in order to have a consistent analysis across human IAV and swine IAV datasets. Sequencing reads were first processed with fastp v.0.23.4 (Chen et al. 2018) to remove reads shorter than 60 nucleotides (nt) or with complexity <30%, trim adapters (with auto-detection) and 3′ poly X runs longer than 10 nt, and perform base correction in overlapped regions. Influenza genomes were assembled and sample-specific consensus sequences inferred using the Iterative Refinement Meta-Assembler (IRMA) FLU v.1.1.4 (Shepard et al. 2016). For each individual, we defined the individual-specific reference sequence to be the consensus sequence from the individual’s earliest sequenced sample. Assembled reads from each influenza segment were re-aligned to the individual-specific reference sequence for that segment using Bowtie 2 v.2.5.4 (Langmead and Salzberg 2012) with default settings (end-to-end mode). LoFreq (Wilm et al. 2012) was used to probabilistically realign reads and call variants relative to individual-specific reference sequences without the use of base-alignment quality scores. Lofreq was also run with permissive settings (no default filter, no minimum base quality, no minimum alternate base quality, significance threshold of 1, Bonferroni factor of 1) to identify the set of variants in the sequencing reads, inclusive of those removed by LoFreq’s filtering protocols. Snakemake v.7.32.4 (Mölder et al. 2021) was used for bioinformatic workflow management. VCF files were processed using Numpy v.2.0.1 (Harris et al. 2020) and Pandas v.2.2.2 (Pandas Development Team 2020) in Python v.3.12.4 (Python Software Foundation 2020).

Variant sites were filtered in R v.4.5.1 (R Core Team 2025) using Tidyverse v.2.0.0 (Wickham et al. 2019) to remove iSNVs that did not exceed a frequency of $\geq 2\%$ at either sampled time point (per pair of adjacent time points), those with a sequencing depth <500 reads at either sampled time point (per pair of adjacent time points), and those located in the first or last 200 nucleotides of a segment. Finally, to prevent variant filtering from giving rise to spurious estimated changes in allele frequencies, we removed any site in which an iSNV was present at $\geq 2\%$ but not called by LoFreq at any of the sampled time points.

Supplemental Fig. S1 shows the human iSNV frequencies arrived at by our pipeline against those from the original human IAV analysis (McCrone et al. 2018). We do not systematically compare our swine iSNV frequencies to those from the original (VanInsberghe et al. 2024) analysis as the original analysis includes reference sequence-specific iSNV filters (e.g. number of mismatches relative to the reference) that are not directly comparable to our analysis with individual-specific reference sequences. Moreover, our IRMA-based genome assembly incorporates a different set of reads as compared with BLAT-based read selection.

### Estimation of effective population size $N_{{E}}$

We estimate the effective population size of within-host human and swine IAV populations by separately interfacing the Beta-with-Spikes model, described above, with human data subsets 1 and 2 and swine data subsets 1 and 2. For both data subsets 1, we set the initial DAF at time $t_{0}$ to have a mean $p_{0}$ given by the observed initial iSNV frequency at the first time point of sampling and a variance given by $v_{0} = mp_{0}(1-p_{0})$. We set the parameter $m$ to 0.004 but we include sensitivity analyses showing that our results are not sensitive to the value of $m$ used. To quantify the probability of observing an allele at a given frequency at the second observation time point, we forward simulate equation (1) under a specified $N_{E}$ from generation $t_{0}$ to generation $t_{k}$, where $k$ denotes the number of viral generations between the first time point and the second time point in a set of paired-time iSNV observations. We assume that a viral generation is 6 h long, based on experimental data (Dou et al. 2017; Einav et al. 2020) and parameter estimates from quantitative models fit to IAV kinetic data (Baccam et al. 2006). As such, the number of viral generations between a pair of iSNV observations is given by $k = (24/6)d = 4d$, where $d$ is the number of days between the observed time points. We include sensitivity analyses of $N_{E}$ under a shorter viral generation time of 3 h and under a longer viral generation time of 12 h.

When the frequency of a focal iSNV at the second time point is above the variant-calling threshold, the probability of observing this data point is simply the probability given by the Beta-with-Spikes distribution evaluated at the observed frequency. When the frequency of a focal iSNV at the second time point falls below the variant-calling threshold, we calculate the probability of observing this data point by using the cumulative density function (cdf), evaluated at the variant-calling threshold. This cdf integrates the probability density function over the frequencies $[0, f_{\mathrm{th}})$, where $f_{\mathrm{th}}$ denotes the variant-calling threshold. The overall log-likelihood for a given $N_{E}$ is given by the sum of the logs of the calculated probabilities across all paired-time observations in data subset 1. For data subsets 2, we first set the initial DAF at time $t_{0}$ to have a mean of $p_{0} = f_{\mathrm{th}}$ and again a variance of $v_{0} = mp_{0}(1-p_{0})$, where we set parameter $m$ to 0.004. The remaining analyses are analogous to those for data subsets 1. Because the first observation time point for each iSNV in data subsets 2 has the iSNV as undetected or falling below 2%, we further consider additional scenarios, namely (i) that the initial frequency of the iSNV is lower than $f_{\mathrm{th}}$ (we consider the initial DAF having a mean of $p_{0} = 0.5\%$ rather than $f_{\mathrm{th}} = 2\%$) and (ii) that the initial frequency of the iSNV is 0% at time $t_{0}$. Under the latter scenario, we consider the scenario of the data subset 2 iSNVs being generated through mutation at a time, i.e. later than $t_{0}$ and capture this scenario by setting the mean of the initial DAF to be either 2% or 0.5% a single viral generation before observation time point 2.

### Assessing model misspecification

To determine whether observed changes in allele frequencies are consistent with a Wright–Fisher model, we first generated mock datasets and applied the Beta-with-Spikes model to these mock data. The mock datasets were generated by setting initial iSNV frequencies at time $t_{0}$ to those in data subset 1 and setting the number of viral generations for those iSNVs to match those in the empirical dataset. For each iSNV, we then forward-simulated the Beta-with-Spikes model to the second observation time point under the maximum-likelihood estimate (MLE) of $N_{E}$ for data subset 1 and sampled an iSNV frequency from the simulated DAF. We generated 1000 of these mock datasets. For each of these mock datasets, we inferred $N_{E}$ and kept track of the log-likelihood value that corresponds to the $N_{E}$ MLE. We further calculated two summary statistics from each of the mock datasets and from the empirical subset 1 datasets: the total absolute change in iSNV frequencies and the proportion of iSNVs lost or fixed at the second observation time point. We then assessed whether the Beta-with-Spikes model could recover the $N_{E}$ value that was used in the simulation of the mock data subsets 1 with log-likelihood values that were similar to those of the empirical data subset 1. Higher log-likelihood values for the mock datasets compared with the maximum log-likelihood value for the empirical data subset 1 could be an indication that the Wright–Fisher model is misspecified and that an alternative evolutionary model may need to be considered to reproduce key features of within-host IAV evolution. We further determined whether the empirical subset 1 datasets yielded values of the summary statistics that were similar to those calculated from the mock datasets. Differences in these summary statistics could be a further indication that the Wright–Fisher model is misspecified.

### Assessing the extent of sampling noise and the possibility of quantifying $N_{E}$

Our application of the Beta-with-Spikes model largely ignores sampling noise. In the most limited sense, sampling noise can result from limited viral material in a sample. It also arises from the number of reads at each iSNV site necessarily being finite. In a broader sense, sampling noise could in principle arise from immigration of viruses into the anatomical site, i.e. sampled. This immigration could stem from other tissues that harbor virus populations that are genetically different from those of the focal anatomical site if there is spatial compartmentalization (Amato et al. 2022; Ganti et al. 2022). Alternatively, this immigration could stem from genetically different viral populations that re-seed an infection from outside of the host, specifically from other nearby hosts that have repeated contact with an infected individual (ecological superinfection). Our application of the Beta-with-Spikes model incorporates sampling noise at the first observation time point in data subsets 1 by incorporating an initial variance around the observed iSNV frequency. However, our likelihood calculation at the second observation time point ignores sampling noise.

To assess the extent of sampling noise and the ability to infer $N_{E}$ in its presence, we temporarily set aside the Beta-with-Spikes model and consider instead the following diffusion approximation for the change in iSNV frequency between two observation timepoints: 


(2) \begin{align*}& \Delta_{q} \approx N(0, q_{0} (1-q_{0}) t / N_{E}) + N(0, q_{0}(1-q_{0})/N_{S})\end{align*}

where $t$ is the number of viral generations between the two timepoints, $q_{0}$ is the frequency of the iSNV at the first observation time point, $N_{E}$ is the viral effective population size, and $N_{S}$ quantifies sampling noise in the broadest sense. A higher value of $N_{S}$ corresponds to less sampling noise. We further define the standardized absolute frequency change of an iSNV as $|\Delta q|/\sqrt{(q_{0}(1-q_{0}))}$. Under the diffusion approximation and in the absence of sampling noise, the expected standardized absolute frequency change of an iSNV is given by $\sqrt{2t / (\pi N_{E})}$. In the absence of genetic drift and in the presence of sampling noise, the expected standardized absolute frequency change of an iSNV is given by $\sqrt{2 /(\pi N_{S})}$. This is also the expected standardized absolute frequency difference of an iSNV, i.e. sampled twice at the same time point (such that $t = 0$). We use these expressions to evaluate whether human and swine data subsets 1 are consistent with a Wright–Fisher model, to quantify the extent of sampling noise in these datasets, and to jointly estimate $N_{E}$ and $N_{S}$.

## Results

### Within-host human influenza A virus effective population sizes are small

Application of the Beta-with-Spikes model to human data subset 1, assuming a viral generation time of 6 h, resulted in a MLE of $N_{E} = 49$ viral particles (95% CI = [28, 84]) (Fig. 2B; Table 1). This result was insensitive to the value of $m$ we used to set the variance of the initial iSNV frequency distribution (Supplemental Fig. S2). Our MLE estimate of $N_{E} = 49$ is consistent with the $N_{E}$ estimate of 32–72 viral particles from McCrone et al. (2020) that used a diffusion approximation. In both cases, the estimated $N_{E}$ values are very small, underscoring the dominant role that genetic drift plays in the evolution of IAV populations within acutely infected humans. Supplemental Fig. S3A shows a sensitivity analysis of this $N_{E}$ estimate assuming instead a 3 h and a 12 h viral generation time. With a 3 h generation time, the MLE of $N_{E}$ increased to 91, whereas with a 12 h generation time, the MLE of $N_{E}$ decreased to 29. This trend of smaller $N_{E}$ estimates at longer generation times is expected, given fewer viral generations between observation time points at long generation times and therefore less “fluctuation opportunity” at the same $N_{E}$. To account for the same iSNV frequency changes between the first and second observation time points, the model therefore needs a lower $N_{E}$ when generation times are assumed to be longer. Despite the larger MLE of $N_{E}$ at a 3 h generation time, this MLE is still very small, again underscoring a strong role for genetic drift in within-human IAV evolution. Finally, we performed a sensitivity analysis to determine whether our iSNV sampling procedure for human data subset 1 impacted our results. When a viral population within a sampled individual had more than one iSNV called at the first observation time point, we had downsampled to a single iSNV. Our downsampling procedure picked the iSNV that had frequency closest to 50% in hopes of reducing the confidence intervals of our $N_{E}$ estimate. We applied the Beta-with-Spikes model to 500 alternative datasets, each instead created by random sampling of an iSNV that was called at a frequency exceeding 2% at the first observation time point. Supplemental Fig. 4A shows that the MLE estimates of $N_{E}$ from these alternative datasets are quantitatively similar to those from human data subset 1. Supplemental Fig. 4B shows that the 95% CI of $N_{E}$ of the alternative datasets are also similar to those of human data subset 1. Our low $N_{E}$ estimates are therefore robust to our choice of iSNV downsampling procedure for data subset 1.

**Table 1:** Effective population size estimates for human data subset 1 and for human data subset 2. For human data subset 2, the table shows $N_{E}$ estimates across a range of different assumptions for the initial frequencies of the iSNVs in this dataset.

**Data subset**	**Initial frequency**	**Time interval between observations**	**MLE of ${N_{E}}$**	**95% CI**
human subset 1	>2% (various)	Original	49	28–84
human subset 2	2%	Original	320	175–600
human subset 2	0.5%	Original	174	118–252
human subset 2	2%	One-generation	47	34–72
human subset 2	0.5%	One-generation	32.5	30–35

### Intrahost single-nucleotide variant frequency changes in human infections are consistent with a Wright–Fisher model of evolution

To determine whether the observed iSNV frequency changes in the human IAV infections were consistent with a basic Wright–Fisher model with a small effective population size, we quantitatively analyzed our mock human data subsets. As expected, application of the Beta-with-Spikes model to these simulated datasets resulted in recovery of $N_{E}$ estimates close to the $N_{E} = 49$ value that was used during their generation (Fig. 2C). Figure 2D shows the log-likelihood values of these simulated datasets, evaluated at the MLE of each dataset’s $N_{E}$, along with that of the empirical human data subset 1. The log-likelihood value of −33.6 for human data subset 1 falls squarely within the distribution of log-likelihoods of the mock datasets. To further examine whether the data from human data subset 1 is consistent with the Wright–Fisher model of evolution, we quantified summary statistics from the Wright–Fisher mock datasets and compared these statistics against those from human data subset 1. The summary statistics we considered were the total absolute frequency change observed in a dataset and the proportion of iSNVs either lost or fixed at the second time point. The values of these statistics, calculated from human data subset 1, fall among the distributions of these statistics from the mock datasets. As such, these findings provide support for the data to have been generated by an underlying model, i.e. consistent with a standard Wright–Fisher model.

### Sampling noise does not have a major impact on intrahost single-nucleotide variant frequency changes in human data subset 1

**Figure 3:**
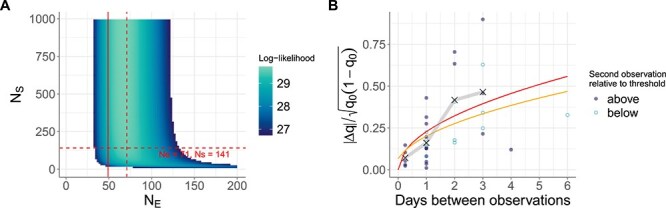
Joint estimation of effective population size ($N_{E}$) and sampling noise ($N_{S}$) for human data subset 1. Cells are shown as colored if within the 95% CI. (A) Estimation of $N_{E}$ and $N_{S}$ from human IAV data subset 1. Vertical solid line shows the MLE estimate of $N_{E} = 49$ from our application of the Beta-with-Spikes model to human data subset 1. Horizontal dashed red line shows the $N_{S} = 141$ estimate from the replicate samples, estimated using equation (2). Dashed vertical red line shows the MLE of $N_{E} = 71$ at $N_{S} = 141$. (B) Drift-standardized absolute changes in iSNV frequencies in human data subset 1 as a function of the number of days between observation time points. The expected value of this summary statistic using the $N_{E}$ estimate of 49 from the Beta-with-Spikes model is shown in red. The expected value of this summary statistic using an $N_{E}$ estimate of 71 and an $N_{S}$ estimate of 141 is shown in yellow. The equation for the expected value of the summary statistic is $\sqrt{\frac{2}{\pi }} \times \sqrt{\frac{t}{N_{E}}\ + \frac{1}{N_{S}}}$.

We next considered the role of sampling noise in impacting our $N_{E}$ estimates in human data subset 1. Using equation (2) and the iSNVs in data subset 1, we jointly estimated $N_{E}$ and the extent of sampling noise $N_{S}$ (Fig. 3A). We found that $N_{E}$ estimates ranged between 30 and 125, regardless of the extent of sampling noise. Indeed, there was a high-likelihood ridge around an $N_{E}$ value of 71 that spanned from low $N_{S}$ (high sampling noise) to high $N_{S}$ (low sampling noise). Our Beta-with-Spikes $N_{E}$ estimate of 49 fell within the 95% confidence region. To determine the extent of sampling noise, we used equation (2) (with the first term on the right-hand side set to 0) and the technical replicates (Supplemental Table S2). We estimated an $N_{S}$ value of 141 using these technical replicates (Fig. 3A dashed horizontal red line). This estimate corresponds to relatively low sampling noise compared with the extent of genetic drift.

Finally, we plotted the drift-standardized absolute change in iSNV frequencies observed in human data subset 1 alongside predictions from the diffusion approximation of the Wright–Fisher model, both with and without sampling noise (Fig. 3B). The data show an increasing trend as a function of time between observations. This empirical trend is captured well with an $N_{E}$ estimate of 49 in the absence of sampling noise. It is also captured well with an $N_{E}$ estimate of 71 and an $N_{S}$ estimate of 141. These results indicate that sampling noise does not have a major impact on iSNV frequency changes in human data subset 1. Moreover, these results provide additional quantitative support for the observed iSNV frequency changes to be driven by a process of genetic drift.

### Human data subset 2 intrahost single-nucleotide variant frequency changes support a small within-host effective viral population size

We now turn to human data subset 2, which comprises pairs of sampling points where the iSNV is called only in the second time point. We use this dataset as additional data to assess support for the very low viral effective population size estimated using human data subset 1. Human data subset 2, however, presents a greater challenge for inference. The issue is that, while the Beta-with-Spikes model easily handles iSNVs that start at an intermediate frequency and then are lost or fixed due to drift, for iSNVs that start rare or absent and then reach observable frequency, the inferred $N_{E}$ is sensitive to the unobserved starting frequency of the iSNV. At one extreme, one can assume that the iSNV was just barely under the calling threshold frequency of 2% at the first sampled time point; such trajectories are shown in Fig. 4A. This minimizes the inferred frequency change and maximizes the time over which that change occurred, and thus represents the minimum amount of drift (maximum $N_{E}$), i.e. consistent with the data. In human data subset 2, with a viral generation time of 6 h, this assumption resulted in an MLE of $N_{E} = 320$ viral particles (95% CI = [175, 600]) (Fig. 4B and Table 1). This estimate of $N_{E}$ is larger than the $N_{E} = 49$ estimated from human data subset 1, but again, this is expected, since this estimate corresponds to the maximum $N_{E}$ estimate, i.e. consistent with the data in human data subset 2. If we instead assume that the iSNVs in human data subset 2 are all present at a frequency of 0.5% at the first observation time point, our $N_{E}$ estimate decreases to 174 (95% CI = 118–252) (Fig. 4C and Table 1). Of course, there is likely variation in initial frequencies of these iSNVs at the first time point of observation. It could even be that some of the iSNVs in human data subset 2 only evolved through the process of mutation *after* the first observation time point. To consider this scenario, we conducted the same analyses as above (with initial frequencies set to 2% and 0.5%) with the first observation time point now assumed to be a single viral generation prior to the second observation time point. Figure 4D shows this assumption when the initial frequencies of the iSNVs are assumed to be 2%. Relative to our previous human data subset 2 results, this assumption resulted in lower $N_{E}$ estimates, with MLE estimates of 47 and 32.5 for initial frequencies of 2% and 0.5%, respectively (Fig. 4E and F; Table 1). These $N_{E}$ estimates are in general agreement with those from human data subset 1. As such, estimates of $N_{E}$ from human data subset 2 are broadly consistent with those from human data subset 1, given reasonable assumptions about iSNV frequencies at the first observation time point and when these iSNVs evolved through the process of mutation.

As we did for human data subset 1, we performed a sensitivity analysis of our $N_{E}$ estimates under different assumptions for the viral generation time. Supplemental Fig. S5 shows log-likelihood values across a range of $N_{E}$ values for an assumed 3 h generation time as well as an assumed 12 h generation time. For both, as in Fig. 3B and C, we considered the initial frequencies of the iSNVs in human data subset 2 to be either 2% and 0.5%. In the case of assuming initial frequency at 2%, the $N_{E}$ estimates decrease from 650 to 160 as the generation time increases from 3 h to 12 h. In the case of assuming initial frequency at 0.5%, the $N_{E}$ estimates decrease from 352 to 84 as the generation time increases from 3 h to 12 h.

### intrahost single-nucleotide variant frequency dynamics in within-host swine influenza A virus populations point to extensive sampling noise, obscuring our ability to estimate $N_{E}$

Figure 5 shows estimates of $N_{E}$ based on the application of the Beta-with-Spikes model on swine data subset 1 assuming a viral generation time of 6 h. The MLE for this dataset is extremely small: $N_{E} = 14.4$ (95% CI = [10.6, 19.2]) (Fig. 5B) and insensitive to the exact value of $m$, i.e. assumed (Supplemental Fig. S2). Supplemental Fig. 3 shows a sensitivity analysis of this $N_{E}$ estimate assuming instead a 3 h and a 12 h viral generation time. Similar to our results from human data subset 1, we find a general trend that the longer the generation time, i.e. assumed, the smaller the MLE of $N_{E}$. Nevertheless, the range of $N_{E}$ estimates across this broad range of plausible viral generation times remains extremely small.

Application of the Beta-with-Spikes model to the mock swine datasets (generated with an $N_{E}$ of 14.4 and an assumed viral generation time of 6 h) resulted in successful recovery of this small $N_{E}$ (Fig. 5C). Interestingly, however, the log-likelihood at the MLE of $N_{E} = 14.4$ for empirical swine data subset 1 was lower than all but 3 mock datasets (0.3%) that were simulated under the Beta-with-Spikes Wright–Fisher model (Fig. 5D), raising initial concern about the consistency of the swine data with the Wright–Fisher evolutionary model. We then quantified total absolute iSNV frequency changes from the mock swine datasets as well as the proportion of iSNVs fixed or lost at observation time 2 from these mock datasets to determine whether the empirical summary statistics from swine data subset 1 fell within the distributions of these summary statistics. Figure 5E and F shows that the empirical summary statistics do indeed fall within the distributions expected from the mock Wright–Fisher simulations with an $N_{E}$ of 14.4.

**Figure 6:**
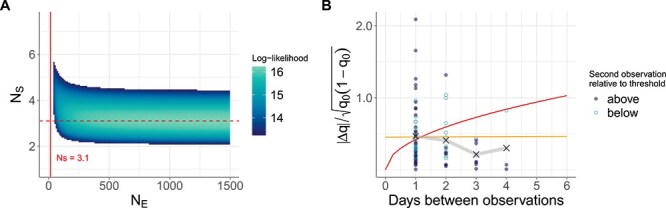
Joint estimation of effective population size ($N_{E}$) and sampling noise ($N_{S}$) for swine data subset 1. Cells are shown as colored if within the 95% CI. (A) Estimation of $N_{E}$ and $N_{S}$ from swine IAV data subset 1. Vertical dashed line shows the MLE estimate of $N_{E} = 14.4$ from our application of the Beta-with-Spikes model to swine data subset 1. Dashed horizontal line shows the $N_{S}$ value of 3.1, corresponding to the maximum-likelihood value of $N_{S}$ along the high-likelihood ridge. (B) Drift-standardized absolute changes in iSNV frequencies in swine data subset 1 as a function of the number of days between observation time points. The expected value of this summary statistic using the $N_{E}$ estimate of 14.4 from the Beta-with-Spikes model is shown with an upwards-curving line in red. The expected value of this summary statistic using an $N_{E}$ estimate of 1500 and $N_{S} = 3.1$ is shown as a horizontal line in yellow.

We then conducted similar analyses to those for human data subset 1 to gauge the extent of sampling noise in swine data subset 1. Figure 6 shows joint estimation of $N_{E}$ and $N_{S}$ for this swine dataset. In contrast to what we found for human data subset 1, we found evidence for a very large amount of sampling noise in swine data subset 1. Indeed, there was a high-likelihood ridge at a value of $N_{S} = 3.1$ that spanned across a broad range of $N_{E}$ values (Fig. 6A). This indicates that the presence of sampling noise impedes our ability to estimate $N_{E}$ for this dataset. In Fig. 6B, we plot the empirical drift-standardized absolute changes in iSNV frequencies from swine data subset 1. We do not observe an increase in this statistic as a function of time between observations, as would be expected under a pure Wright–Fisher model. Indeed, expected drift-standardized absolute changes in iSNV frequencies under a Wright–Fisher model with $N_{E} = 14.4$ do not reproduce the empirical (flat) trend, indicating that the simple Wright–Fisher model of genetic drift does not recover patterns in the swine data. However, a model with only sampling noise (with $N_{S} = 3.1$) does reproduce these empirical patterns (Fig. 6B). These results indicate that sampling noise is extensive in the swine data. This impedes our ability to estimate $N_{E}$ using this dataset. Given these results, we forego further analyses of the swine data using swine data subset 2.

## Discussion

Several previous studies have underscored the prominent role that genetic drift plays in shaping the evolutionary dynamics of IAV populations in acutely infected individuals (Dinis et al. 2016; Debbink et al. 2017; McCrone et al. 2018, 2020; Bendall et al. 2024; VanInsberghe et al. 2024). Here, we reanalyzed two previously published datasets (one human IAV dataset and one swine IAV dataset) to quantify the extent of genetic drift acting on within-host IAV populations in natural infections. Consistent with a previous study that estimated the effective viral population size from the same set of acutely infected humans to be $N_{E} = 32-72$ (McCrone et al. 2020), we here similarly found evidence of a very small $N_{E}$ of around 50 viral particles. The consistency of these findings is reassuring, given that the previous study used a diffusion approximation that can break down at small $N_{E}$ values. Our estimate was instead derived from the Beta-with-Spikes model that has been shown to provide accurate estimates for both small and large $N_{E}$ values (Tataru et al. 2015). There are several other differences between the approach taken by McCrone et al. (2020) and the one we used here. McCrone et al. (2020) used all of the iSNV frequency data at once and jointly estimated $N_{E}$ and the mutation rate $\mu $. We instead partitioned the data into two subsets. The first subset included the iSNVs that were detected above the variant-calling threshold at the first observation time point and the second included the iSNVs where iSNVs were identified above the variant-calling threshold at the second observation time point but were not called at the first observation time point. This partitioning allowed us to avoid estimation of the mutation rate, which should impact not only sites with observed iSNVs but also those that do not show evidence of polymorphism. Our approach and the approach taken by McCrone et al. (2020) are complementary, and the consistency of our $N_{E}$ estimates underscores the robustness of the results.

Recent work by Bendall et al. (2024) that analyzed the evolutionary dynamics of seasonal IAV populations within acute human infections also estimated $N_{E}$. The estimates of $N_{E} = 176-284$ arrived at in this study are considerably higher than those estimated with the previous human IAV dataset from the same group (McCrone et al. 2018, 2020) that we also examined here. We do not know the reason for the difference in $N_{E}$ estimates between these two human IAV datasets. One possibility, and our favored hypothesis, is that the difference has to do with the difference in the variant-calling threshold applied to the datasets. In McCrone et al. (2018), a 2% threshold was applied, whereas in Bendall et al. (2024), a 0.5% threshold was applied. To test this hypothesis, we reanalyzed human data subset 1 at a variant-calling threshold of 0.5% (instead of our original 2%). Supplemental Fig. S6 shows that the MLE of $N_{E}$ increases from our original estimate of 49–123 with a 0.5% variant-calling threshold. It also shows that for this human IAV dataset, the MLE of $N_{E}$ stabilizes at our estimate of 49 only at variant-calling thresholds that exceed 1.6%. This confirms that the difference in variant-calling thresholds could cause the difference in inferred $N_{E}$ values. However, if all the alleles are evolving according to Wright–Fisher dynamics, changing the range of allele frequencies used to estimate $N_{E}$ should not impact the $N_{E}$ point estimate (just the breadth of the confidence intervals). One way that different groups of alleles could give different $N_{E}$ estimates is if one group is enriched for spurious variants that are not truly evolving. In this case, spurious variants are expected to be more common at lower variant-calling thresholds. In other contexts, it has been observed that such spurious low-frequency variants can be consistent across timepoints and thus tend to bias estimates of $N_{E}$ upwards (Martin and Koelle 2021). This would suggest that the lower $N_{E}$ values inferred here and in McCrone et al. (2018) may be more accurate than the higher values inferred by Bendall et al. (2024). However, Bendall et al. (2024) did perform a benchmarking analysis to determine how low they could go with their variant-calling threshold.

If Bendall et al. (2024)’s $N_{E}$ estimate was inflated due to the presence of spurious variants at this low 0.5% variant-calling threshold, they may have also overestimated positive selection. This is because the approach used by Bendall et al. (2024) sequentially estimates $N_{E}$ and then selection coefficients. At large values of $N_{E}$, dramatic changes in iSNV frequencies have to invoke selection, as genetic drift cannot explain these changes. If $N_{E}$ is actually smaller than estimated, then a subset of the dramatic changes in iSNV frequencies will, therefore, no longer need to invoke selection, as genetic drift could explain the changes. Overestimation of $N_{E}$ due to a too-low variant-calling threshold might therefore explain why this previous work identified a number of synonymous iSNVs to be under positive selection. In any case, it would be interesting to gauge the robustness of the $N_{E}$ estimate from the Bendall et al. (2024) dataset under a higher variant-calling threshold of 2%.

We would like to underscore that the $N_{E}$ estimates currently in the literature, including the ones provided here, are from natural infections. Effective population sizes can also be estimated from experimental infections that are sampled longitudinally. Recent estimates of $N_{E}$ for IAV populations in experimentally infected humans are also very small (on the order of 20 viral particles), considerably smaller than the $N_{E}$ estimates of $\sim $200 for IAV populations in ferrets that were experimentally infected using the same viral inoculum (Ferreri et al. 2026). Intriguingly, effective population sizes for norovirus GI.1 populations sampled from fecal specimens of experimentally infected humans have also been estimated to be small, on the order of 50–61 (Tohma et al. 2025), underscoring the role that genetic drift also plays in the evolution of this virus. We are not currently aware of any within-host $N_{E}$ estimates for contact animals from experimental transmission studies, although visual inspection of barcoded IAV dynamics from a recent study by Holmes et al. (2025) indicates that, interestingly, genetic drift does not appear pronounced in these populations. $N_{E}$ estimation using the Beta-with-Spikes model could, in principle, be done using barcoded virus data, with a single barcode or combination of barcodes chosen to represent a focal allele. Alternatively, other, more powerful statistical approaches could be used in the case of barcoded viral data.

Beyond estimation of within-host effective viral population sizes, we here simulated mock datasets and used these datasets to help us address the question of whether the empirical datasets are consistent with the classic Wright–Fisher model of evolution. Through this analysis, we found that the iSNV frequency changes observed in the human IAV dataset were consistent with the Wright–Fisher model. In contrast, we found that the evolutionary dynamics observed in the swine IAV dataset could not be robustly reproduced by the classic Wright–Fisher model. Instead, we found evidence for a large amount of sampling noise (in the broadest sense) in this dataset. This result is critical and indicates that future studies need to focus on determining what other processes might be contributing to within-swine IAV evolution. Ecological superinfection is a likely possibility, given the high proportion of pigs that were infected at the county fair and the extent of dual H3N2–H1N1 infections observed (VanInsberghe et al. 2024). While we limited our analyses here to what appeared to be single infections, it is very likely that the infected pigs repeatedly contacted other infected pigs. This would allow for re-inoculation, invalidating the assumption of a closed viral population within each animal. Spatial compartmentalization is another possibility, given extensive empirical evidence that IAV populations are spatially structured across respiratory tract tissues (Gallagher et al. 2018; Amato et al. 2022; Ganti et al. 2022; Ferreri et al. 2025). The classic Wright–Fisher model used here also assumes a constant population size, which is clearly not the case for acute respiratory virus infections, with viral population sizes changing by several orders of magnitude over a time period of under 50 viral generations. Cellular coinfection and the impacts of this coinfection on viral progeny production is also not captured in any way under a Wright–Fisher model. Relaxation of some of these limitations, e.g. to consider within-host compartmentalization and potentially time-varying effective population sizes, will allow us to not only better dissect the roles of different evolutionary processes that occur within hosts but also to better understand the processes that guide the path from mutation generation to viral spread at the population level.

## Supplementary Material

clean_withinhostIAV_Ne_revision_supplemental_veag021

table_S1_veag021

table_S2_veag021

table_S3_veag021

## Data Availability

All inference code is available on GitHub at: https://github.com/koellelab/IAV_beta_with_spikes_Ne.
