# Supplementary material for: Genetic drift acts strongly on influenza virus populations within acute human infections but is obscured by other factors within acutely infected swine
Source: Virus Evol. 2026 Apr 1;12(1):veag021. doi: 10.1093/ve/veag021 (PMC13100903; doi:10.1093/ve/veag021)
Supplement: clean_withinhostIAV_Ne_revision_supplemental_veag021 [file clean_withinhostiav_ne_revision_supplemental_veag021.pdf]

Genetic drift acts strongly on influenza virus populations within acute human infections but is obscured by other factors within acutely infected swine

Supplemental Material

Yike Teresa Shi[1], Michael A. Martin[2], Daniel Weissman[3], Katia Koelle[1,4,*]

1 Department of Biology, Emory University, Atlanta, GA, USA
2 Department of Pathology, Johns Hopkins School of Medicine, Baltimore, MD, USA
3 Department of Physics, Emory University, Atlanta, GA, USA
4 Emory Center of Excellence for Influenza Research and Response (CEIRR), Atlanta GA, USA

[*]katia.koelle@emory.edu

Supplemental Figures

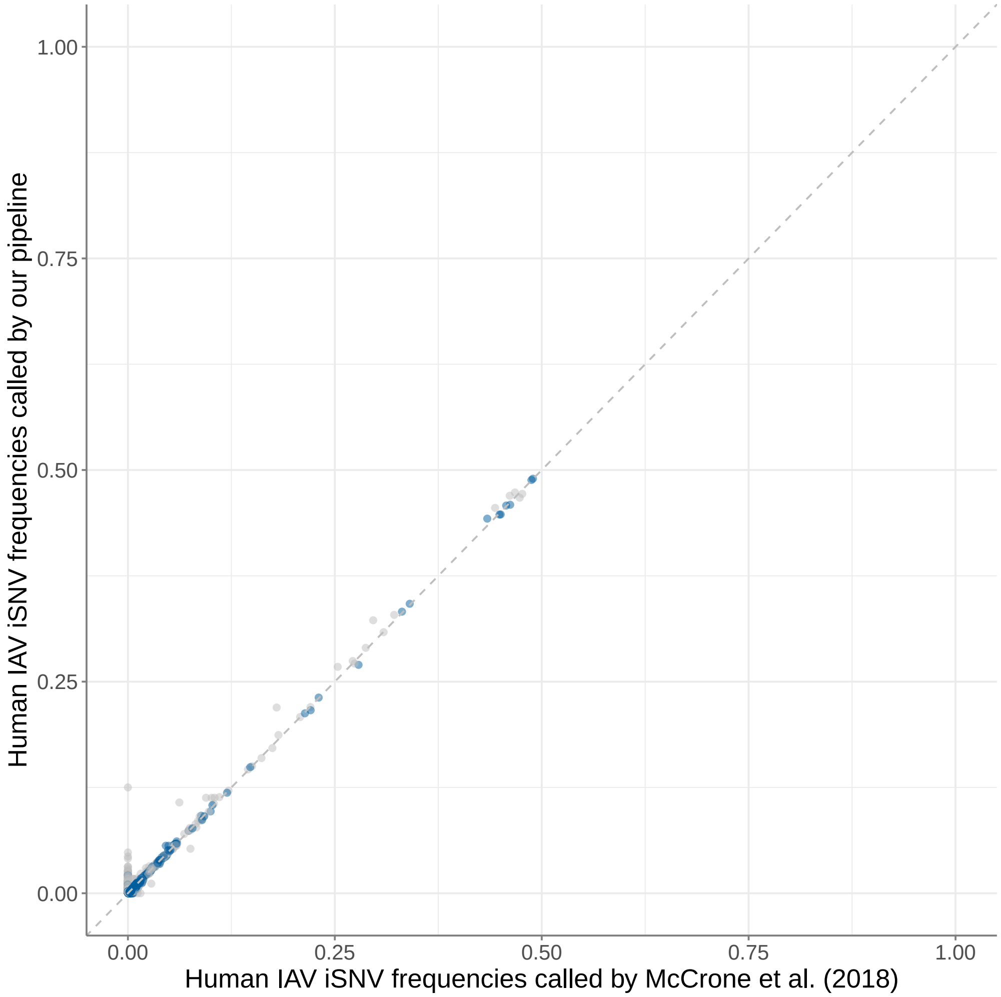

Figure S1. Comparison between human IAV iSNV frequencies called in the original analysis by McCrone et al. (2018) *eLife* and those called by our pipeline (detailed in the *Methods* section). Our pipeline and the McCrone et al. (2018) pipeline yield highly similar iSNV frequencies (dots along the $x = y$ diagonal). Points are colored by whether samples were sequenced in replicate or not. Blue dots show iSNVs without technical replicates. Grey points show iSNVs from technical replicates. In the case of replicate sequencing, McCrone et al. selected iSNV frequencies from the replicates on a per-iSNV basis whereas we selected iSNV frequencies on a per-sample basis (using the sample with higher sequencing depth).

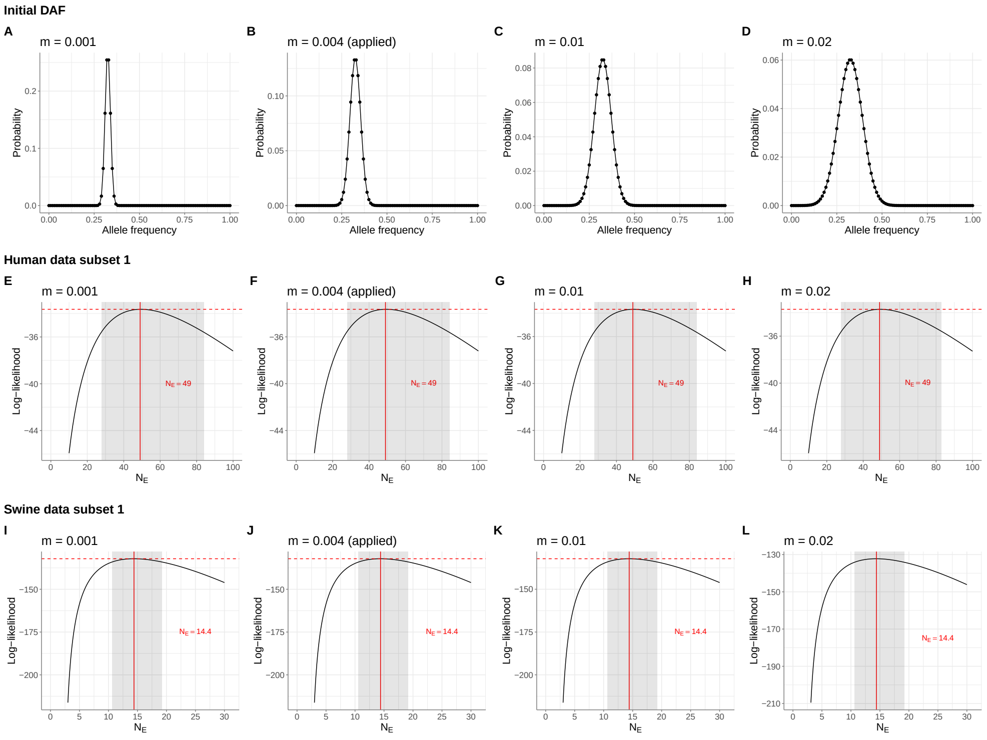

Figure S2. Sensitivity analyses to assess the impact of m on the estimation of N_E. Four different values for m are considered: $m = 0.001$ (column 1), $m = 0.004$ (column 2; reproduced from the main manuscript); $m = 0.01$ (column 3), and $m = 0.02$ (column 4). (A-D) The initial DAF for an iSNV initially observed at a frequency of $p_0 = 0.325$ under various different assumptions for the value of m. The variance of the DAF is given by $v_0 = mp_0(1 - p_0)$. (E-H) Sensitivity analyses for the value of m for human data subset 1. (I-L) Sensitivity analyses for the value of m for swine data subset 1.

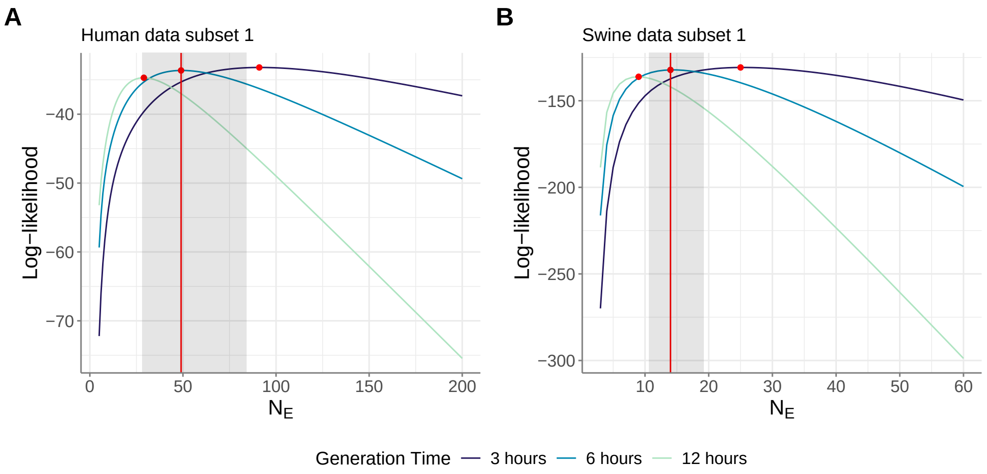

Figure S3. Generation time sensitivity analysis. (A) Generation time sensitivity analysis of human IAV data subset 1. (B) Generation time sensitivity analysis of swine IAV data subset 1. Log-likelihood curves assuming a 3-, 6-, and 12- hour viral generation time are shown using purple, cyan, and green lines, respectively. Maximum likelihood estimates of N_{E} are shown using red dots. The MLE of N_{E} with the 95% confidence interval calculated with a fixed 6-hour generation time is shown with a solid red line and surrounding shaded region.

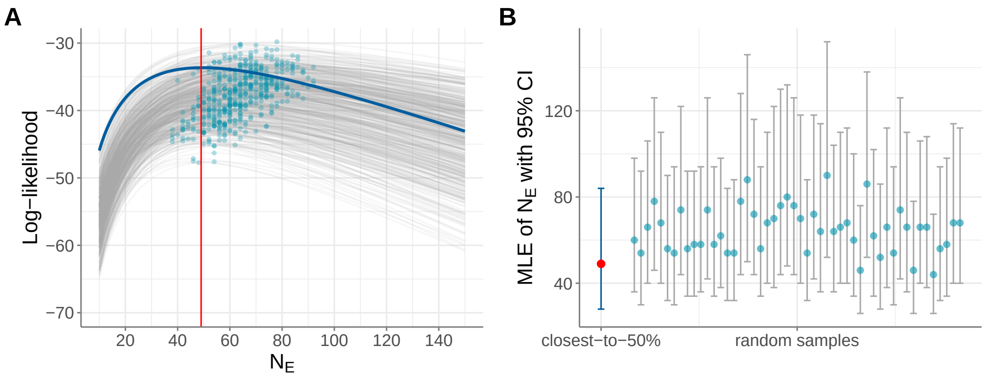

Figure S4. Estimates of within-host IAV effective population size from 500 random samples of datasets with iSNVs present in the first sampled timepoint using a variant-calling threshold of 2%. Unlike in the analyses of the human and swine data subsets 1 in the main manuscript, we here downsample these sets of iSNVs to one iSNV per individual by selecting one random iSNV that is above variant-calling threshold at the first observation time point per person. 500 random samples are generated using this method for the human data as well as for the swine data. (A) Calculated log-likelihood values across a range of effective population sizes for human data subset 1 and 500 random samples. The log-likelihood curves calculated from the 500 random samples are shown in gray. The maximum likelihood estimates (MLEs) of N_{E} calculated from the random samples are shown in light blue. The log-likelihood curve calculated from human IAV data subset 1 obtained by the closest-to-50% method is shown in dark blue. The MLE of N_{E} calculated from human IAV data subset 1 is shown with a vertical red line. (B) MLEs and the corresponding 95% confidence intervals of N_{E} for 50 random samples, compared to the MLE and 95% confidence interval calculated from human IAV data subset 1. The MLEs and the corresponding 95% confidence intervals of N_{E} calculated from the 50 random samples are shown with light blue points and gray bars, respectively. The MLE and the corresponding 95% confidence interval of N_{E} calculated from human data subset 1 are shown with a red point and a dark blue bar, respectively.

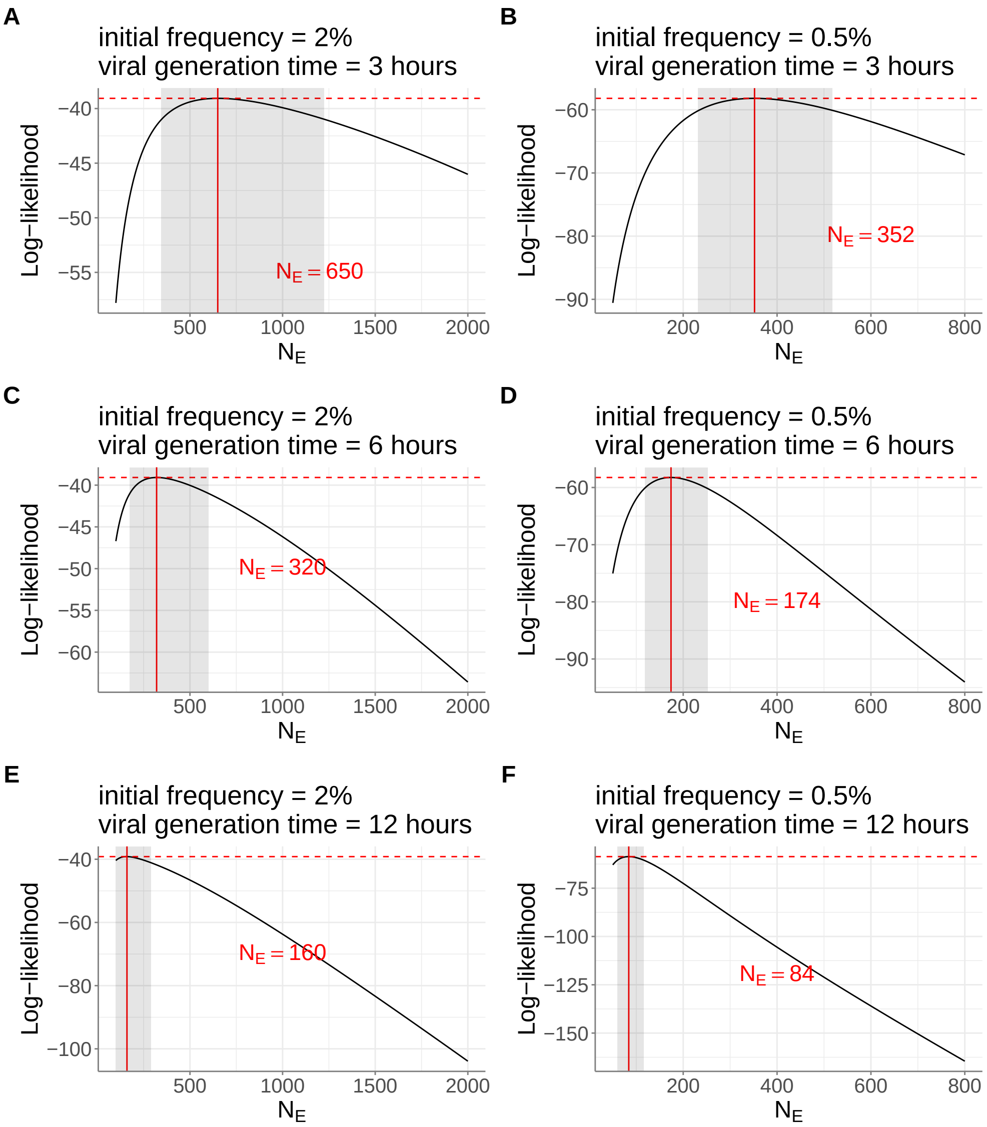

Figure S5. Viral generation time sensitivity analysis for human IAV data subset 2. We calculated log-likelihood values for N_E using human data subset 2 assuming 3- and 12-hour viral generation times. The 6-hour viral generation time results are reproduced from Figure 4 panels (B) and (C). In our sensitivity analyses, we considered both 2% and 0.5% initial frequencies at the first time point.

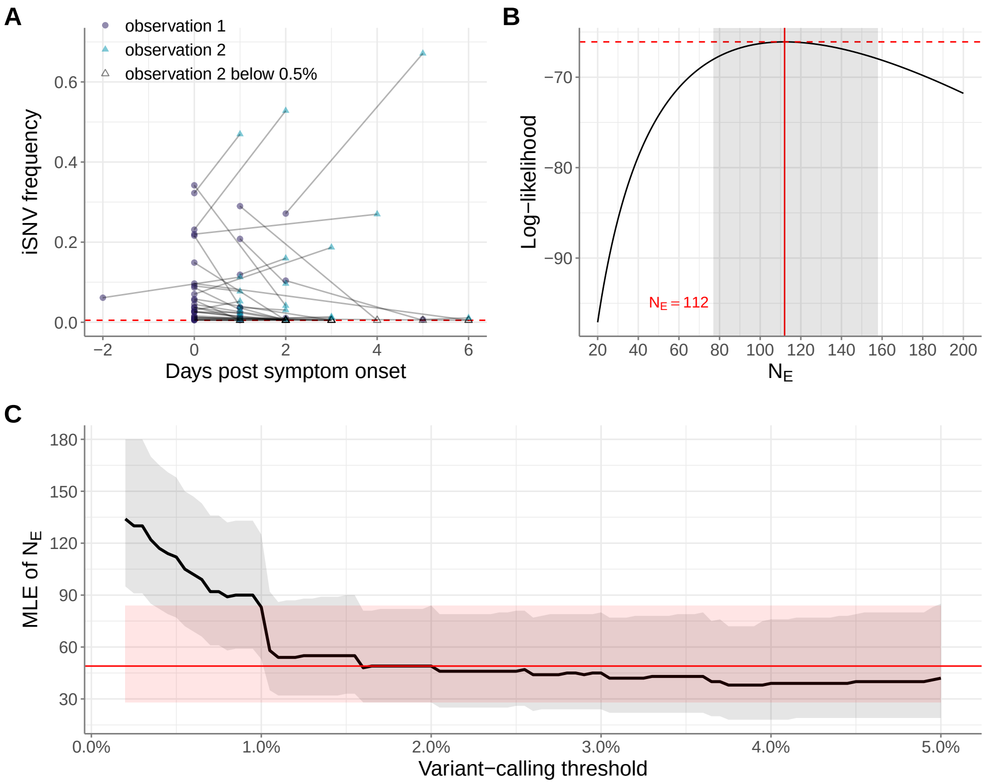

Figure S6. Variant-calling threshold sensitivity analysis for N_E values estimated from iSNVs in the McCrone et al. (2018) data where the first sampled time point had a detected frequency above a given variant-calling threshold. (A) Allele frequency changes between the first observation time point and the second observation time point using a variant-calling threshold of 0.5%. Allele frequencies are plotted by day of symptom onset of the infected individual. The red dashed line shows the variant-calling threshold of 0.5%. Allele frequencies at the second observation time point that fall under this threshold are shown at the threshold. In comparison to Figure 2A, this panel shows a greater number of iSNVs due to the inclusion of additional iSNVs with frequencies at the first observation time point that fell between 0.5% and 2%. (B) Calculated log-likelihood values across a range of effective population sizes using the data shown in panel (A) and assuming a viral generation time of 6 hours. Solid red line shows the maximum likelihood estimate (MLE) of N_E. Dashed red line shows the log-likelihood value for the MLE of N_E. The shaded region shows the 95% confidence interval around the MLE of N_E. The MLE of $N_E = 112$ using this iSNV dataset exceeds the MLE of $N_E = 49$ using human data subset 1 that uses a 2% variant-calling threshold (Figure 2B). (C) Variant-calling threshold sensitivity analysis. The observation pairs are re-selected for each variant-calling threshold to ensure the inclusion of lower-frequency iSNVs when applicable. At each variant-calling threshold, iSNVs were still downsampled to one iSNV per indvidual by selecting the iSNV that had a frequency closest to 50% at the first time point. The black line and the gray shaded region show, respectively, the maximum likelihood estimation (MLE) of N_E and the corresponding 95% confidence interval calculated under each variant-calling threshold. Solid red line and red shaded region shows the maximum likelihood estimate (MLE) of N_E and the corresponding 95% confidence interval calculated using variant-calling threshold of 2% (as in the main text).
